# Morbid Impulses

**Published:** 1880-01

**Authors:** Horatio R. Bigelow


### MORBID IMPULSES.

BY HORATIO R. BIGELOW, M.D.

In an article written for the Cincinnati *Medical News*, in May, 1874, I offered the following explanation of the morbid impulse:

"When the impulse becomes dominant, asserting itself despite the will, then it is that the person is pronounced insane. The mere existence of the fixed idea, so long as it be controlled by volition, is in no wise an abnormality. When the hemispherical cells cease to react upon each other harmoniously, when an idea prolongs its tension so as to 'tyrannize over the understanding, and become an absorbing entity,' illusions and delusions result. A man in this condition of mental erythism, acting under a delusion, would not be amenable to law, only in so far as his confinement in a proper asylum would be demanded. The *modus operandi* by which an idea becomes excited and active is this: The necessary external stimulus applied to the sensory ganglia is expressed outwardly, as pleasure or disgust, while the residua furnish to the well-balanced mind the stimulus which was necessary to excite the particular idea in one of the numerous cortical cells. Just what stimulus was needed, and just what idea would obtain from its application, are the lessons stamped on the mental growth by the experience of generations. The nervous action may become weakened by the vicious transmission of heredity, or the integrity of the nervous vitality of the centres may be upset by injurious practices."

A more precise observation has forced the belief upon me that a morbid impulse, which is always dominant and may not be controlled by the will, never originates *de novo*, but is the result of previous family instability. The underlying predisposition to the various conditions of mental erythism may always be found in a transmitted tendency of heredity, or, in women, in uterine disorders and misplacements. The hypochondria incident to acute dyspepsia is often the offspring of eccentricity (so-called) in either the father or the mother, and may, in turn, become the parent of a more pronounced form of mental unsoundness in the next generation. Each one, in his life's history, may remember the existence of a transitory impulse, which, had it been realized outwardly in action, would have occasioned shame and disgrace. But such occasions only become matters of legitimate legal inquiry when they are offered in extenuation of crime. An influential consideration which must always be a prominent factor in the ultimate diagnosis is the social position of the patient. The commission of a criminal act by a person whose previous record has been untarnished, who has never been vicious or immoral, whose education has been elevating, and whose associations such as to tend to develop and strengthen the better sentiments of human nature, is much more apt to be caused by disease than would be a similar realized impulse in one whose constant acquaintance with crime had lowered the tone and brought into prominence the brutal passions.

The law differentiates in the two instances with equity and good sense, consigning the one to an insane asylum and the other to prison. Morbid introspection, or the constant consideration of an impulse to commit an offense, will sometimes become so overmastering that the victim, recognizing the imminent danger to himself and others in a weakened will power, will request to be put under surveillance. Such a case, and one of great interest, is fully described by M. Dagonet, in the *Journal de Medecine Mentale*, 1869, p. 317. It is also worthy of note that all the cases cited in the literature of mental disease as instances of morbid impulse have occurred in the middle or higher grades of society. The advance of civilization, rather than its absence or retardation, exerts a marked influence in their development. The greed of gain, the fluctuations of the money market, the exciting condiments of sumptuous tables, the sensual and demoralizing literature and art which delight the æsthetic young people of the period, are the necessary evils of our day and generation. Ignorance will foster superstition, debase the intellect and weaken the mental growth, but it will not disrupt the harmonious intercommunication of perception and will. Very rarely, if ever, has it been the case that a dominant impulse has obtained among the ignorant. When the existence of this condition of mental erythism is urged in the extenuation of crime, it should be the duty of the physician to inquire minutely into the inherited tendencies of the prisoner, to seek for parental eccentricities, to weigh well the previous mental states, the social position and early training ; and should it be a woman, to examine carefully for uterine flexions. Many women have been confined in asylums for acute and chronic insanity, who have recovered almost immediately upon the correction of a mal-placed uterus. Disorders of the digestive apparatus may be the exciting cause in a person so predisposed by reason of a transmitted taint.

The relation of the morbid impulse to crime is an intimate one. The abuse of the plea in criminal courts should not blind our eyes to its frequent existence. Just when the court may make a discrimination, and differentiate between a crime committed with a calm and sane deliberation, and others committed on the impulse of the moment, or from the predominating assertion of a morbid impulse, is a matter of frequent and interesting medico-legal inquiry. The theory of mania transitoria, urged with so much ability and success in the Reynolds and McFarlane trials, could not bear the test of intellectual inquiry. While such states of mental unsoundness are incident to epilepsy and cerebral congestion, giving rise to transitory mania, no essential and primary disease of this kind is known in neurology. Yet a crime committed by a person who himself was a victim to epilepsy, or whose antecedents had been epileptic, might be condoned with propriety upon such a plea. The heat of passion occasioned by wrongs, imaginary or actual, does occasion cerebral hyperæmia, but such a plea could not be accepted in equity, by any court, as palliative of an offense committed. The immediate antecedent and subsequent mental condition of the prisoner, in relation to the crime, had been normal. He had no inherited disease, and the passion was self-caused and might have been controlled. No man may take the law into his own hands. To urge the plea of mania transitoria in such a case, because a condition of cerebral hyperæmia did not obtain, is to stultify scientific medicine. The desire of redress for wrongs inflicted is natural and general. The Christian fortitude to bear our ills with serenity—to suffer long and be kind—is rare. The constant contemplation of our troubles may lead to morbid mental states; but, except in rare instances, the criminal impulse may always be controlled by the will; and even where the will has become weakened we are always conscious of its approach, and our relations to society then demand that we should seek sanitary intervention. A morbid impulse beyond the control of the will never manifests itself without previous warning. A criminal act perpetrated under these conditions, is to be taken cognizance of by the law, only in so far as the patient is criminal in not surrounding himself with necessary protection against the outburst. In advanced stages, even for such negligence, a man may not be accountable, as a will sufficiently weakened to fail in controlling any manifestation presupposes that it is equally weak in all things, and is not, therefore, capable of realizing its own insufficiency. Such a painful condition is true insanity, and should be treated as such.—*Med. and Surg. Reporter.*

